# High baseline levels of PD-L1 reduce the heterogeneity of immune checkpoint signature and sensitize anti-PD1 therapy in lung and colorectal cancers

**DOI:** 10.1038/s41419-025-07471-w

**Published:** 2025-03-04

**Authors:** Peng Fan, Ziwei Qi, Zhenhua Liu, Shanshan Wang, Ying Wang, Jiajie Kuai, Naidong Zhang, Wei Xu, Songbing Qin, Eleonora Candi, Yuhui Huang

**Affiliations:** 1 Cyrus Tang Medical Institute, State Key Laboratory of Radiation Medicine and Prevention, Collaborative Innovation Center of Hematology, Soochow University, Suzhou, China; 2 Cancer Institute, Suzhou Medical College, Soochow University, Suzhou, China; 3 Department of Experimental Medicine, TOR, University of Rome “Tor Vergata”, Rome, Italy; 4 National Center of Technology Innovation for Biopharmaceuticals, Suzhou Biomedical Industry Innovation Center, Suzhou, China; 5 Department of Radiotherapy, the First Affiliated Hospital of Soochow University, Suzhou, China; 6 Department of Radiotherapy, Yancheng First Hospital, Affiliated Hospital of Nanjing University Medical School, Yancheng, China; 7 Institute of Clinical Pharmacology, Key Laboratory of Anti-Inflammatory and Immune Medicine, Ministry of Education, Anhui Collaborative Innovation Center of Anti-inflammatory and Immune Medicine, Anhui Medical University, Hefei, China; 8 New Drug Biology and Translational Medicine, Innovent Biologics Inc., Suzhou, China

**Keywords:** Immune evasion, Cancer immunotherapy

## Abstract

Immune checkpoint blockade (ICB) therapy only induces durable responses in a subset of cancer patients. The underlying mechanisms of such selective efficacy remain largely unknown. By analyzing the expression profiles of immune checkpoint molecules in different statuses of murine tumors, we found that tumor progression generally randomly upregulated multiple immune checkpoints, thus increased the Heterogeneity of Immune checkpoint Signature (HIS) and resulted in immunotherapeutic resistance. Interestingly, overexpressing one pivotal immune checkpoint in a tumor hindered the upregulation of a majority of other immune checkpoint genes during tumor progression via suppressing interferon γ, resulting in HIS-low. Indeed, PD-L1 high-expression sensitized baseline large tumors to anti-PD1 therapy without altering the sensitivity of baseline small tumors. In line with these preclinical results, a retrospective analysis of a phase III study involving patients with non-small cell lung cancer (NSCLC) revealed that PD-L1 tumor proportion score (TPS) ≥ 50% more reliably predicted therapeutic response in NSCLC patients with baseline tumor volume (BTV)-large compared to patients with BTV-small. Notably, TPS combined with BTV significantly improved the predictive accuracy. Collectively, the data suggest that HIS reflects the dynamic features of tumor immune evasion and dictates the selective efficacy of ICB in a tumor size-dependent manner, providing a potential novel strategy to improve precision ICB. These findings highlight the application of ICB to earlier stages of cancer patients. The integration of PD-L1 with BTV may immediately improve patient stratification and prediction performance in the clinic.

## Introduction

Immune evasion is a hallmark of cancer [1]. As tumors grow larger, they develop stronger capabilities to escape detection by the immune system [2, 3]. Tumors can evade host immunosurveillance through various mechanisms, including the accumulation of suppressive immune populations [4, 5], the downregulation of the molecules related to antigen presentation [6, 7], hypoxia-associated immunosuppression [8, 9], the production of immune-inhibitory components [10, 11], and the upregulation of immune checkpoints [12–15]. Accordingly, various therapeutic strategies have been developed to counter these mechanisms and induce antitumor immunity, such as therapies targeting immunosuppressive cells, adoptive T cell therapy, cancer vaccines, immunomodulatory cytokines, and immune checkpoint blockade (ICB) therapy [16–19].

Currently, ICB therapy stands out as the only approach demonstrating durable antitumor efficacy across a variety of cancer types in the clinic, underscoring the dominant role of immune checkpoints in solid tumor immune evasion. Even though, only a small fraction of patients demonstrates long-term survival benefits from such therapy [19–21]. The underlying mechanisms that mediate the selective efficacy of ICB remain incompletely understood. Given that tumor progression often involves the simultaneous upregulation of various immune checkpoints, such as programmed death-1 (PD1), programmed death ligand-1 (PD-L1), cytotoxic T-lymphocyte antigen 4 (CTLA4), CD200, T cell immunoglobulin mucin 3 (TIM3), lymphocyte-activation gene 3 (LAG3), poliovirus receptor-related immunoglobulin domain-containing protein (PVRIG) [19, 22], it raises a fundamental question: why is blocking one immune checkpoint pathway, i.e., PD1/PD-L1, sufficient to induce tumor regression in the presence of many other immunosuppressive factors commonly found in the tumor microenvironment (TME)?

To address the question, we compared the profiles of immune checkpoint expression and response to ICB in small and large murine tumors and proposed a new concept: the heterogeneity of immune checkpoint signature (HIS). Our preclinical data revealed that small tumors typically exhibited low levels of immune checkpoint expression, resulting in HIS-low and sensitive to anti-PD1 therapy. In contrast, large tumors often exhibited stochastic upregulation of multiple immune checkpoint molecules, leading to HIS-high and resistance to anti-PD1 therapy. Additionally, large tumors could enhance their immune evasion ability via overexpressing a few pivotal immune checkpoints. Since PD1/PD-L1 blockade therapy is a widely used ICB therapy, and the tumor proportion score (TPS) of PD-L1 has been used to stratify potential responders for this therapy [23, 24], we tested this scenario by overexpressing PD-L1 in tumor cells. Interestingly, elevated PD-L1 levels hindered the upregulation of other immune checkpoint genes by suppressing interferon γ (IFNγ) in large, but not small, tumors, resulting in decreased HIS and increased sensitivity to anti-PD1 therapy. Moreover, a retrospective analysis of a phase III study (NCT03607539) involving patients with non-small cell lung cancer (NSCLC) showed that PD-L1 TPS ≥ 50% more reliably predicted the response to anti-PD1 combination therapy in patients with baseline large tumors compared to those with small ones. Together, these results suggest that the selective efficacy of ICB is associated with the characteristics of HIS, providing a novel strategy to improve patient stratification and precision ICB therapy.

## Materials and methods

### Study design

This study aimed to unveil the mechanisms of the selective efficacy of ICB. Preclinical murine tumor models were designed to investigate the relationship among the baseline status of HIS, PD-L1 expression, tumor size, and to test the hypothesis that HIS dictates the outcomes of ICB. The retrospective clinical analysis explored the translational potential of HIS in the patient stratification and precision ICB therapy.

Pilot studies were conducted to determine the parameters and sample sizes. Sample numbers (n) and the number of experimental replicates for each experiment were indicated in the figure legends. No data were excluded from any studies. Mice were randomly assigned to experimental groups sequentially based on their tumor sizes, which were ranked using an “S-curve” distribution to ensure that all groups had similar baseline tumor sizes. Investigators were not blinded to sample allocation, data acquisition, or data analysis (with the exception of image quantification); however, objective measurements and standardized procedures minimize potential bias.

### Animal models

C57BL/6 female mice (6–8 weeks old) were purchased from the Shanghai Laboratory Animal Center (Shanghai, China). *Ifng*^−/−^ C57BL/6 mice were purchased from the Jackson Laboratory. FVB mice were bred in the specific pathogen-free (SPF) animal facility of Soochow University. All mice were kept in micro isolator cages under conditions with a 12-hour light-dark cycle, at a temperature of 21–23°C, and humidity ranging from 35% to 55%, within the SPF animal facility of Soochow University.

### PD-L1 overexpression and cell culture

The MCA38 tumor cell line was purchased from the American Type Culture Collection (Manassas, VA, USA). The lung carcinoma cell line (LAP0297) was generated by Dr. Peigen Huang at Massachusetts General Hospital (Boston, USA) [25]. For PD-L1 overexpression, the murine *Pd-l1* gene was cloned and integrated into the lentiviral pCDH-CMV-MCS-EF1α-copGFP expression vector (Catalog: CD511B-1, System Biosciences). Parental MCA38 and LAP0297 cells underwent transduction with the retroviral vector to establish stable cell lines overexpressing PD-L1 (MCA38-PD-L1, LAP-PD-L1). Cell lines transduced with pCDH vectors (MCA38-pCDH, LAP-pCDH) were employed as controls. The levels of PD-L1 expression in the tumor cell lines were determined by qPCR and flow cytometry. All cell lines were cultured in Dulbecco’s modified Eagle’s medium (DMEM; Gibco, USA) supplemented with 10% heat-inactivated fetal bovine serum (Gibco, USA) and 1% penicillin-streptomycin (Gibco, USA) and maintained in a humidified incubator (37 °C, 5% CO_2_). Regular checks for Mycoplasma contamination were performed using a PCR Mycoplasma Test Kit (Catalog: K0103, HuaAn Biotechnology).

### Tumor growth and treatments

To assess the impact of tumor burden on anti-PD1 therapy efficacy, LAP0297 (LAP) tumor cells (1 × 10^5^ cells) were subcutaneously injected into the right flank of FVB mice. When the tumors reached diameters of 3.0–4.0 mm and 5.0–6.0 mm, the mice were randomly allocated to different treatment groups. Subsequently, they were intraperitoneally administered either anti-PD1 antibody (Innovent Biologics Co., LTD, 5 mg/kg) or isotype-matched IgG_2a_ (Catalog: #BE0089, Bio X Cell, 5 mg/kg) every 3 days for a total of 3 doses. For MCA38 colorectal tumors, 2 × 10^5^ cells were subcutaneously inoculated into the right flank of female C57BL/6 mice. When the tumors reached diameters of 4.0–5.0 mm and 7.0–8.0 mm, the mice were randomly divided into two groups and received either isotype IgG or anti-PD1 therapy, respectively. Treatment response was assessed based on the final tumor volume, with stable or reduced volumes indicating response and increased volumes indicating non-response.

To investigate the impact of HIS levels on anti-PD1 therapy, mice bearing LAP-PD-L1 or LAP-pCDH lung tumors were treated with anti-PD1 or IgG for 3–4 times, at a dosage of 5 mg/kg. Mice bearing small or large MCA38-pCDH and MCA38-PD-L1 colorectal tumors were treated with anti-PD1 or IgG for 3 times at a dosage of 0.2 mg/kg or for 4 times at a dosage of 2 mg/kg, respectively. In addition, mice bearing large MCA38-pCDH or MCA38-PD-L1 tumors mice were treated with an anti-CTLA4 (catalog: BE0164, clone 9D9, 5 mg/kg) or control IgG_2b_ (catalog: BE0086, clone MPC11, 5 mg/kg) antibodies every 3 days for a total of 4 doses. Both treatments were given intraperitoneally at a volume of 100 μl. Tumor size was monitored by measuring the length and width with a caliper and the tumor volume (mm^3^) was estimated according to the following formula: (long axis) × (short axis)^2^ × π/6.

In vivo, depletion of T cells was performed following established procedures [26–28]. Briefly, mice bearing MCA38-PD-L1 tumors were randomly divided into 7 groups. Some of the groups received intraperitoneal injections of 200 μg isotype-matched control antibody IgG2a, anti-CD8 monoclonal antibody (Catalog: #BE0004-1, Bio X Cell), anti-CD4 monoclonal antibody (Catalog: #BE0003-1, Bio X Cell), or both, administered on days 15, 17, and 22. Some of the groups were intraperitoneally injected with anti-PD1 or IgG for 3 times at a dosage of 2 mg/kg on days 16, 19, and 22. The efficiency of T-cell depletion was assessed using flow cytometry.

For anti-IFNγ antibody treatment, mice bearing MCA38-pCDH tumors with diameters of 4.0–5.0 mm were randomly divided into two groups and received intraperitoneal injections of isotype control rat IgG_1_ (catalog: BE0088, clone HRPN, 100 μg/mouse) or anti-IFNγ antibody (catalog: BE0055, clone XMG1.2, 100 μg/mouse), every three days for a total of 3 dosages.

### RNA sequencing

**Fig. 1:**
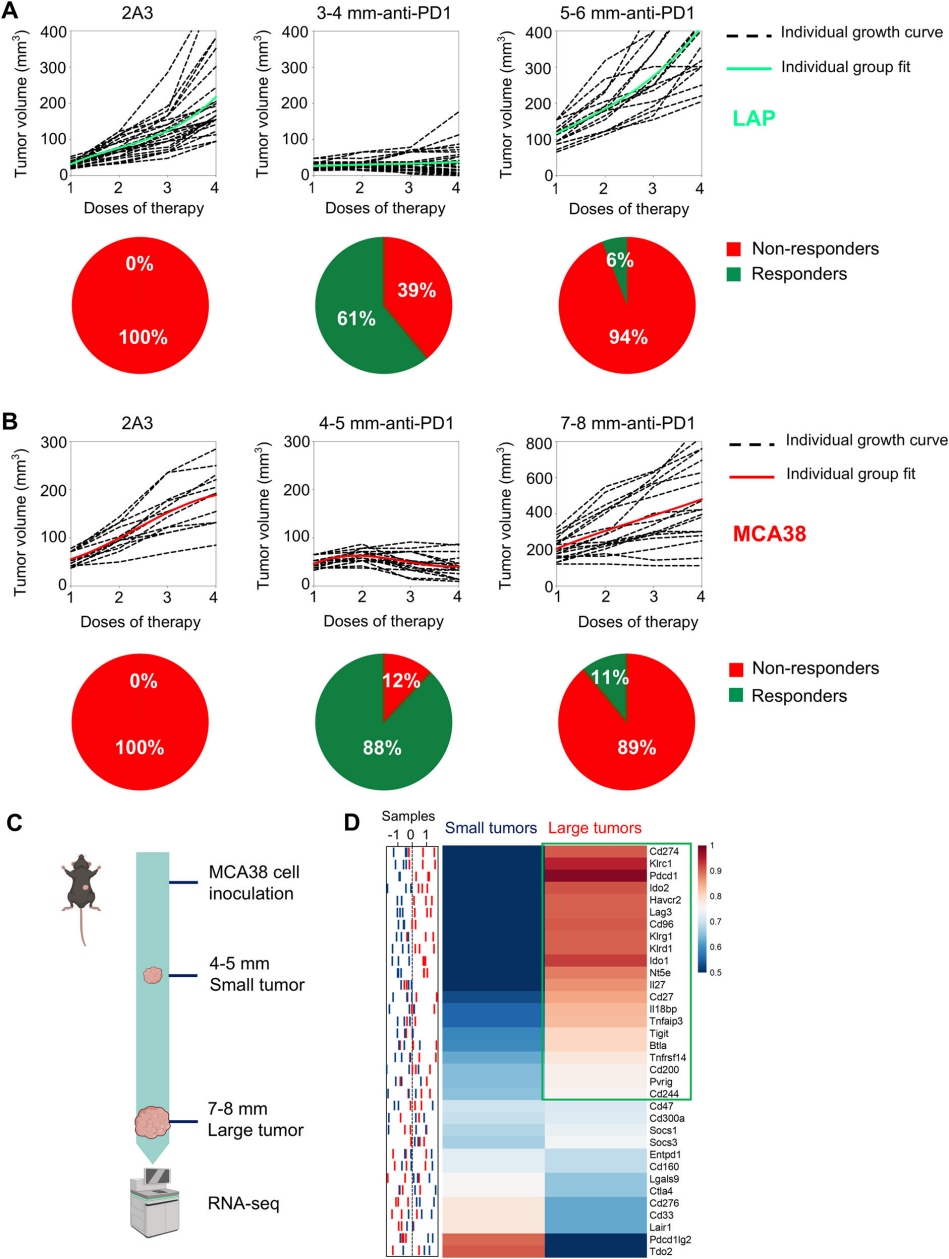
The efficacy of anti-PD1 therapy diminished as the heterogeneity of the immune checkpoint signature (HIS) increased with tumor progression. **A** When LAP0297 lung tumors reached 3.0–4.0 mm (small tumor) or 5.0–6.0 mm (large tumor) in diameter, mice were randomly assigned to two groups and received either isotype IgG or anti-PD1 therapy. Tumor growth curves and the response rates were depicted. **B** When MCA38 tumors reached 4.0–5.0 mm (small tumor) or 7.0–8.0 mm (large tumor) in diameter, mice were randomly divided into two groups and received isotype IgG or anti-PD1 therapy, respectively. Tumor growth curves and the response rates were shown. Data represent 2 independent experiments with similar results in (**A** and **B**). **C** Schematic of the experimental workflow. MCA38 tumors were harvested at small (4.0–5.0 mm) and large (7.0–8.0 mm) sizes for RNA sequencing. **D** The sample distribution plot (left) showed the profiles of immune checkpoint gene expression in small (blue) and large (red) MCA38 tumors derived from RNAseq data, with the corresponding heatmap (right) presented as group-level representations. Data include three biological replicates (*n* = 3).

RNA-seq was outsourced to Gene Denovo Biotechnology Co (Guangzhou, China). In brief, total RNA extraction was carried out utilizing a TRIzol reagent kit (Invitrogen, Carlsbad, CA, USA) following the manufacturer’s protocol. The quality of the extracted total RNA was evaluated using an Agilent 2100 Bioanalyzer (Agilent Technologies, Palo Alto, CA, USA) and RNase-free agarose gel electrophoresis. Subsequently, eukaryotic mRNA was selectively enriched from the total RNA pool using oligo(dT) beads. The enriched mRNA underwent fragmentation into shorter fragments through the application of a fragmentation buffer. These fragments were then reverse transcribed into complementary DNA (cDNA) using the NEBNext Ultra RNA Library Prep Kit for Illumina (NEB #7530, New England Biolabs, Ipswich, MA, USA). The resulting double-stranded cDNA fragments were subjected to a series of preparatory steps, including end repair, addition of an A base, and ligation to Illumina sequencing adapters. The ligated fragments were purified with AMPure XP Beads (1.0X) and then subjected to size selection by agarose gel electrophoresis and PCR amplification. The generated cDNA library was sequenced using an Illumina NovaSeq6000 sequencer. The heatmap and GSEA analyses were performed via the online platform “OMICSHARE” (https://www.omicshare.com/). The sample distribution plot was generated by standardizing the values of gene expression FPKM (Fragments Per Kilobase of exon model per Million mapped fragments). The standardized values were then visualized using GraphPad Prism (version 9) to create the distribution plot. The RNAseq data were used to quantify HIS. Briefly, the FPKM values of 34 commonly studied immune checkpoint genes, such as PD1, PD-L1, CTLA4, LAG3, TIM3, and TIGIT, were extracted as shown in Fig. 1D. The FPKM metric normalizes gene expression levels based on transcript length and sequencing depth, enabling accurate comparisons across samples. To classify tumors into different HIS categories, the profiles of immune checkpoint genes from early-stage, ICB-sensitive tumors were used as the baseline. For each gene, the fold change (FC) in expression was calculated by dividing its mean FPKM value in baseline large tumors by the corresponding mean value from baseline small tumors. Tumors were classified as HIS-high if ≥10 immune checkpoint genes exhibited an FC ≥ 2.0, and as HIS-low if ≤5 genes met this criterion.

### Quantitative real-time PCR

Total messenger RNA (mRNA) from cells or tumor tissues was isolated using a MicroElute Total RNA kit (Omega, Norcross, GA, USA), followed by cDNA synthesis using a RevertAid First Strand cDNA Synthesis Kit (Thermo Scientific, Minneapolis, MN, USA). Quantitative real-time PCR was conducted using a LightCycler 480 SYBR Green I Master Mix (Roche, Boston, MA, USA) in a High Throughput Quantitative PCR LightCycler 480 (Roche). The comparative threshold cycle method was utilized to calculate differences in gene transcription (fold change) by normalization to the reference gene *β-actin*. Primer sequences are listed in Table S1.

### Flow cytometric analysis

Flow cytometry analysis was conducted on a Gallios flow cytometer (Beckman) following previously established procedures [26, 28]. Briefly, tumor tissues were diced into small fragments and incubated at 37 °C for 45 min in DMEM supplemented with collagenase type 1 A (1.5 mg/mL, Sigma-Aldrich), hyaluronidase (1.5 mg/mL, Sigma-Aldrich), and deoxyribonuclease I (DNase I; 20 U/mL, Sigma-Aldrich). Subsequently, this enzymatic digest was filtered through 70 μm nylon cell strainers to obtain single-cell suspensions. For cultured cells, cells were detached using 0.25% trypsin to obtain single-cell suspensions. FcγR was blocked utilizing a rat anti-mouse CD16/CD32 antibody (BD Pharmingen). Subsequently, single cell suspensions were stained, washed and resuspended in a cold flow buffer composed of 1% bovine serum albumin and 0.1% NaN_3_ in PBS.

Intracellular staining for IFNγ was performed as described previously [26, 27]. In brief, 0.1% Brefeldin A Solution (Catalog: 420601, eBioscience) was added to the cell suspensions and incubated for 4 h at 37 °C. Then, the cells were fixed and permeabilized by using a Fixation/Permeabilization Solution Kit (Catalog: 554714, BD Bioscience) per the manufacturer’s instructions, followed by intracellular staining. Analysis of flow cytometry data was carried out utilizing Kaluza software (version 1.3). The following reagents were used: CD45-BV421 and CD11b-BV510 (all from BD Pharmingen); CD8-FITC, CD8-PE, CD8-PE-Cy7, CD4-PE, CD4-AF700, CD69-PE-Cy7, CD44-APC, CD25-APC, CD25-APC-Cy7, Gr1-APC, Gr1-APC-Cy7, PD-L1-PE-Cy7, PD1-APC, PD1-APC-Cy7, IFNγ-APC and 7-amino-actinomycin D (7-AAD) (all from BioLegend).

### Human patient samples

The dataset originated from a previously published phase III clinical trial (ClinicalTrials.gov: NCT03607539). This is a multicenter, randomized and double-blinded trial to compare the efficacy of chemotherapy (pemetrexed and platinum) with placebo versus its combination with sintilimab (an anti-PD1 antibody) in patients with nonsquamous NSCLC [29]. The PD-L1 TPS data were extracted from the clinical trial records. Specifically, tumor PD-L1 expression was evaluated using the PD-L1 IHC 22C3 pharmDx assay (Agilent Technologies) on either archival tumor specimens or fresh biopsy samples, which were centrally analyzed at Covance in Shanghai, China.

### Response and survival analysis

Patient responses to the therapy were assessed based on the criteria outlined in the Response Evaluation Criteria in Solid Tumor version 1.1 (RECIST 1.1). Responders were defined as individuals whose tumors shrank by more than 30% compared to baseline and maintained tumor control for a minimum of 24 weeks. Non-responders included patients whose tumors did not shrink or displayed enlargement within 24 weeks after initial shrinking. Overall survival (OS) for patients who were alive or lost to follow-up was censored at the time last seen alive. The R “survminer” package was used to generate survival curves for visualization.

### CECT image analysis

CECT image data collection followed the Chinese health industry standard “CT examination operating procedures” (WST391-2012). Patients were positioned in the supine position on the bed, with their arms raised, and instructed to hold their breath during the scanning process. The scanning parameters included 120 kV voltage, automatic mA settings ranging from 100 to 300 mA, and a reconstructed layer thickness of no more than 5 mm. The iodine injection dosage administered ranged from 300 to 450 mg iodine/kg body weight, with an injection rate of 3–4 mL/s. Scanning during the arterial phase was performed ~20–30 s after the injection. A physician with 10 years of experience in medical imaging and radiation therapy used 3D Slicer software (Version 4.11.0) to outline the volume of interest (VOI). The tumor outlines were semi-automatically sketched using a threshold range of −50 to 200 Hounsfield Units (HU). The volume measurement module was utilized to automatically calculate the baseline tumor volumes (BTVs) based on the selected VOI contour. The cut-off value of BTVs with the highest Youden index was determined based on the receiver operating characteristic (ROC) curves.

### Predictive model and performance assessment

Two logistic regression models were developed to predict patient response to anti-PD1 combination therapy based on area under the curves (AUCs). The first model analyzed the predictive power of PD-L1 TPS, while the second model examined the joint prediction of PD-L1 TPS and BTV. Both models were trained using a shared dataset, with TPS and/or BTV as independent variables and immunotherapy response outcomes as the dependent variable. To enhance predictive accuracy, stricter TPS thresholds of ≥25%, ≥50%, and ≥75% were adopted for analysis. BTV cutoff values were determined using the ROC curves derived from the entire cohort of 129 patients, with the optimal Youden index defining the threshold. Tumors above this cutoff were classified as BTV-large, while those below were classified as BTV-small. The predictive models were constructed using logistic regression in MedCalc statistical software. Separate logistic regression equations were developed for each TPS threshold:

$${\rm{For}}\; {\rm{TPS}}\ge 25 \% :{\rm{Logit}}=-0.7155+0.9993\times {\rm{BTV}}+1.876\times {\rm{TPS}}\,25$$

$${\rm{For}}\; {\rm{TPS}}\ge 50 \% :{\rm{Logit}}=-0.7347+1.241\times {\rm{BTV}}+2.398\times {\rm{TPS}}\,50$$

$${\rm{For}}\; {\rm{TPS}}\ge 75 \% :{\rm{Logit}}=-0.4042+1.122\times {\rm{BTV}}+2.329\times {\rm{TPS}}\,75$$

Model performance was assessed using the AUC of ROC curves. Statistical comparisons between models were conducted using DeLong’s test to evaluate the effectiveness of the predictive models.

### Statistical analysis

Statistical analysis was performed using GraphPad Prism (version 9). The number of animals and replicates was indicated in each figure or the legend. Mean values were compared using unpaired, two-tailed Student’s *t*-tests for comparisons between two independent groups, and one‐way ANOVA was adopted when comparing more than two groups. Two‐way ANOVA was applied to compare the means of more than two groups at multiple levels of two categorical variables. Differentially expressed genes (DEGs) were conducted using DESeq2 software, applying a threshold of |log2 (fold change)| > 1 and an FDR < 0.05. OS was estimated by the Kaplan‒Meier method, and differences were determined by the log-rank test. Hazard Ratio (HR) and 95% confidence interval (CI) were computed using the Cox proportional hazard model. ROC curves and AUCs were used to evaluate the prediction power. All data are presented as the mean ± standard error of the mean (SEM). Differences were considered statistically significant when *P* < 0.05.

## Results

### HIS increases with tumor progression, resulting in resistance to anti-PD1 therapy

Clinical studies have consistently shown that higher baseline tumor burden is associated with a poorer prognosis following ICB therapy [30–34]. To recapture this clinical phenomenon, we treated LAP0297 lung tumor-bearing mice with an anti-PD1 antibody when tumor size reached 3.0–4.0 mm (small tumors) and 5.0–6.0 mm (large tumors) in diameter, respectively. In baseline small tumors, anti-PD1 therapy significantly inhibited tumor growth, with ~61% of mice showing durable responses. By contrast, when treatments were initiated in baseline large tumors, less than 6% of mice displayed a durable response (Fig. 1A). Similarly, in baseline small and large MCA38 colorectal tumors, anti-PD1 therapy induced durable responses in 88% and 11% of mice, respectively (Fig. 1B). These data show that anti-PD1 efficacy decreases with increasing baseline tumor burden. To elucidate the underlying mechanisms, we performed bulk RNA sequencing (RNA-seq) analysis of baseline small and large tumors (Fig. 1C). We identified 34 immune checkpoint genes that showed significant differences between the two types of tumors: 21 genes were upregulated in baseline large tumors, while only 9 genes were upregulated in baseline small tumors (Fig. 1D). These data suggest that tumor progression increased HIS, which may lead to resistance to anti-PD1 therapy.

### Overexpressing PD-L1 reduces HIS in baseline large tumors by suppressing IFNγ

**Fig. 2:**
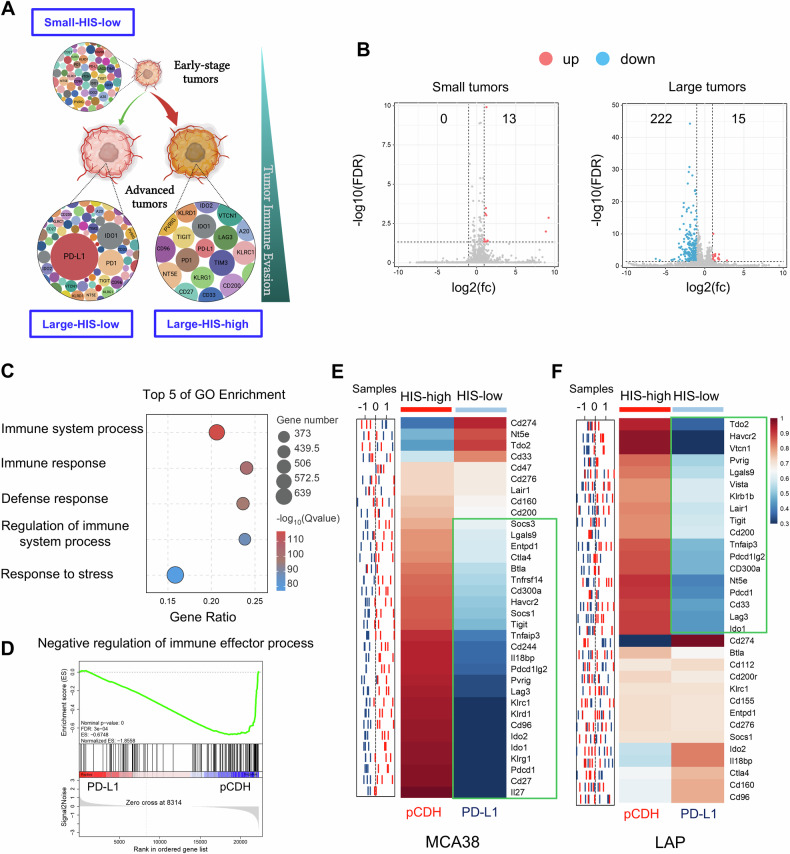
PD-L1 high-expression reduced HIS with tumor progression. **A** Schematic diagram of tumor immune evasion mediated by HIS: immune checkpoints express at low levels during tumor initiation (HIS-low), and tumor progression requires stronger immune evasion capabilities via either randomly upregulating multiple immune checkpoints (HIS-high) or overexpressing a few immune checkpoints (HIS-low). **B** Volcano plot displaying differentially upregulated (pink dots) and downregulated (blue dots) genes, comparing small or large pCDH and PD-L1 tumors, with a threshold of |log2-fold change (fc)| >1 and FDR < 0.05. **C** Top 5 GO terms of downregulated gene enrichment in large MCA38 tumors. **D** GSEA indicating negative enrichment of genes related to negative regulation of immune responses in large MCA38-PD-L1 tumors compared to control MCA38-pCDH tumors. *P* values were automatically determined by GSEA v.3.0. ES, enrichment score; NES, normalized enrichment score; FDR, false discovery rate. **E**, **F** The sample distribution plot (left) illustrated the profiles of immune checkpoint gene expression for pCDH (red) and PD-L1 (blue) in MCA38 and LAP tumors based on RNAseq data. The corresponding heatmap (right) showed group-level representations, with three biological replicates (*n* = 3) for MCA38 tumors and five biological replicates (*n* = 5) for LAP tumors.

To further explore the impacts of the levels of HIS on ICB efficacy, we need to develop tumor models with either low or high HIS. Tumor progression accompanies with stronger immune evasion, which could be realized by two distinct mechanisms: randomly upregulating multiple immune checkpoints simultaneously (multiple-IC) and overexpressing a minority of immune checkpoints (dominant-IC) (Fig. 2A). Most of advanced tumors will evade host immunosurveillance through multiple-IC as shown in Fig. 1. Considering that PD1/PD-L1 blockade therapy is a primary ICB and that higher PD-L1 levels are associated with better ICB efficacy, we postulated that HIS-low and HIS-high tumor models could be established by manipulating PD-L1 expression. We then established stable overexpression of PD-L1 in MCA38 colorectal (MCA38-PD-L1) and LAP0297 lung (LAP-PD-L1) tumor cells (Supplementary Fig. S1A, B). Both PD-L1-overexpressing MCA38-PD-L1 and control MCA38-pCDH tumors exhibited comparable growth rates during tumor initiation phase (typically within the first week following tumor cell inoculation). Subsequently, a remarkable acceleration in tumor growth kinetics was observed in the MCA38-PD-L1 group during later stage (Supplementary Fig. S2A). Similar results were observed in the LAP tumor model (Supplementary Fig. S2B). The data show that elevated PD-L1 levels promote the growth of late-stage tumors rather than early-stage ones, indicating that overexpressing PD-L1 promotes advanced tumor immune evasion.

We then performed RNA-seq analysis on pretreatment small and large tumors from MCA38-pCDH and MCA38-PD-L1 groups. In baseline small tumors, merely 13 genes were identified as upregulated without any downregulated gene, based on a threshold of |log2 (fold change)| > 1 and FDR value < 0.05 in MCA38-PD-L1 tumors compared to MCA38-pCDH tumors. In contrast, in baseline large tumors, 15 genes were upregulated, whereas 222 genes were downregulated in MCA38-PD-L1 tumors relative to MCA38-pCDH tumors (Fig. 2B). These results suggest that PD-L1 overexpression primarily suppresses gene transcription in baseline large tumors. Further analysis of the altered genes in the context of baseline large tumors revealed a significant enrichment of terms linked to immune processes (Fig. 2C). Additionally, gene set enrichment analysis (GSEA) revealed a consistent negative enrichment of genes associated with the negative regulation of immune response processes in large tumors with PD-L1 overexpression compared to their control counterparts (Fig. 2D). Thus, we analyzed the alterations in immune checkpoints between MCA38-PD-L1 and MCA38-pCDH tumors. Interestingly, apart from the *Pd-l1* gene, 81.8% (27/33) of immune checkpoints were downregulated in MCA38-PD-L1 tumors compared to MCA38-pCDH tumors (Fig. 2E). This was further validated by qPCR analysis, which showed that the *Pd-l1* gene was upregulated while all the other tested immune checkpoints were downregulated in MCA38-PD-L1 tumors compared with MCA38-pCDH tumors (Supplementary Fig. S3). Similarly, qPCR analysis data showed that, aside from the upregulation of the *Pd-l1* gene, 56.3% (18/32) of immune checkpoints were downregulated, while 25.0% remained unchanged in baseline large LAP-PD-L1 tumors compared to LAP-pCDH tumors (Fig. 2F). Taken together, these data demonstrate that multiple immune checkpoint genes were upregulated in baseline large control tumors, while a majority of immune checkpoint genes were downregulated in baseline large tumors with PD-L1 overexpression. Therefore, baseline large tumors with PD-L1 overexpression and its size-matched control tumors can serve as the HIS-low and HIS-high tumor models, respectively.

**Fig. 3:**
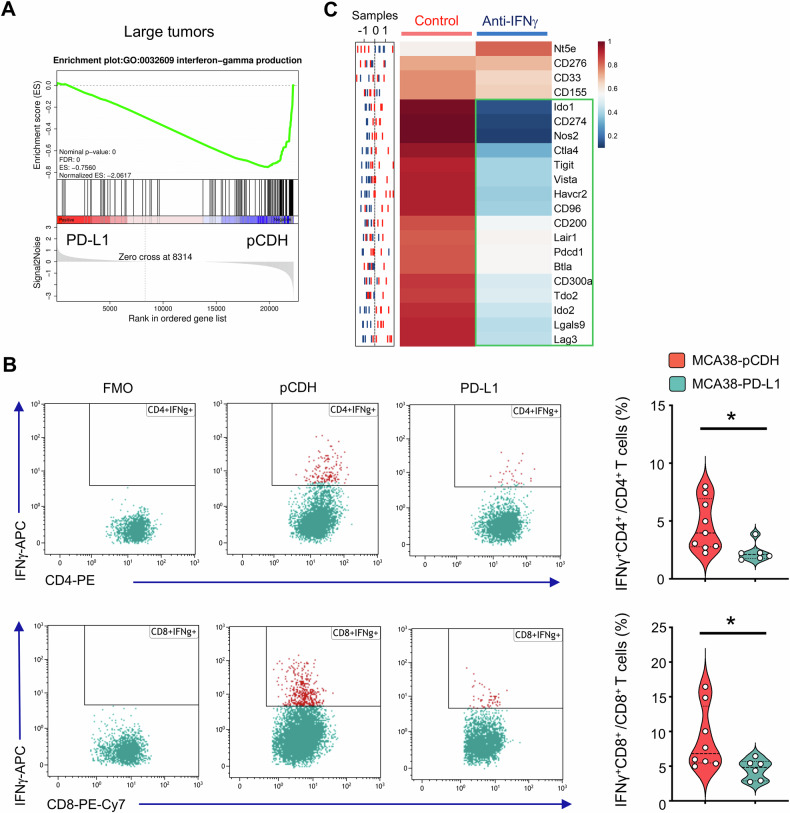
Overexpressing PD-L1 reduced IFNγ production, resulting in decreased HIS in baseline large tumors. **A** GSEA showing negative enrichment of genes related to IFNγ production in large tumors of MCA38-PD-L1 compared to control MCA38-pCDH. *P* values were automatically determined by GSEA v.3.0. ES, enrichment score; NES, normalized enrichment score; FDR, false discovery rate. **B** IFNγ production in intratumoral CD4^+^ and CD8^+^ T cells. FMO, fluorescence-minus-one. Data were analyzed by two-tailed Student’s *t*-tests; each dot represents one individual tumor sample, and data are shown as the mean ± SEM. Data represent 2 independent experiments with similar results. **C** The sample distribution plot (left) showed the profiles of immune checkpoint gene expression in MCA38-pCDH treated with anti-IFNγ antibody (blue) or IgG control (red) based on RNAseq data. The corresponding heatmap (right) displayed group-level results, with five biological replicates (*n* = 5).

Next, we sought to investigate the underlying mechanisms by which PD-L1 overexpression impedes the upregulation of other immune checkpoint genes. GSEA analysis revealed a consistent negative enrichment of genes associated with IFNγ production in large tumors with PD-L1 overexpression compared to their control counterparts (Fig. 3A). Flow cytometric analysis also showed that PD-L1 overexpression reduced IFNγ production in intratumoral CD4^+^ and CD8^+^ T cells (Fig. 3B). Furthermore, in *Ifng* knockout mice, the growth curves of MCA38-pCDH and MCA38-PD-L1 tumors were comparable (Supplementary Fig. S4). Moreover, neutralization of IFNγ led to a reduction in HIS levels in MCA38-pCDH tumors (Fig. 3C). Taken together, the data show that overexpressing PD-L1 hinders the upregulation of other immune checkpoint genes in baseline large tumors by suppressing IFNγ production, resulting in a HIS-low phenotype.

According to the above data, we classified the baseline small tumors, regardless of PD-L1 expression levels, as “small-HIS-low”, baseline large pCDH tumors as “large-HIS-high”, and baseline large tumors with PD-L1 overexpression as “large-HIS-low” in the following studies.

### The profiles of HIS guide toward a more effective ICB therapy

**Fig. 4:**
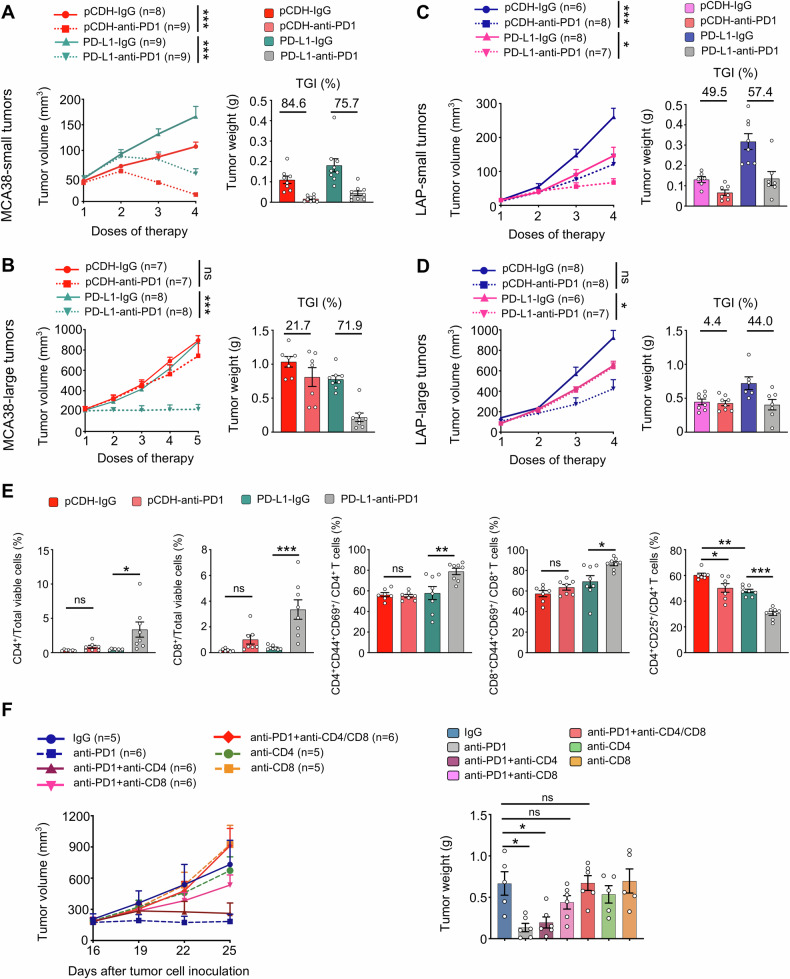
HIS-low tumors were sensitive to anti-PD1 therapy, while HIS-high tumors were resistant. The growth curves and tumor growth inhibition (TGI) of MCA38-pCDH and MCA38-PD-L1 tumors treated with either IgG or an anti-PD1 antibody when tumor sizes reached 4.0–5.0 mm (**A**) and 7.0–8.0 mm (**B**) in diameter, respectively. The growth curves and TGI of LAP-pCDH and LAP-PD-L1 tumors treated with IgG or an anti-PD1 antibody when the sizes of tumors reached 3.0–4.0 mm (**C**) and 5.0–6.0 mm (**D**) in diameter, respectively. Treatments were administered intraperitoneally at the indicated dose regimes every 3 days. **E** Control MCA38-pCDH or MCA38-PD-L1 tumors were treated with anti-PD1 or isotype IgG antibodies when tumors reached 7.0–8.0 mm and continued every 3 days. The percentages of intratumoral CD4^+^ T cells, CD8^+^ T cells, CD4^+^CD25^+^ Tregs, CD4^+^CD44^+^CD69^+^ T cells, and CD8^+^CD44^+^CD69^+^ T cells were analyzed by flow cytometry. (**F**) The growth curves and weights of MCA38-PD-L1 tumors were monitored after treatment with various antibody regimes (IgG, anti-PD1, anti-CD4, anti-CD8) administered as indicated in the figure every 3 days. Treatments were initiated when tumors reached 7.0–8.0 mm in diameter. Each dot represents one individual tumor sample. Data are shown as the mean ± SEM. Significance was determined by two-way ANOVA. Data represent two (**A**, **B**, **E**, **F**) or three (**C**, **D**) independent experiments with similar results.

To investigate the influence of HIS levels on ICB efficacy, we evaluated the response to anti-PD1 therapy across various levels of HIS. In small-HIS-low tumors of MCA38-pCDH and MCA38-PD-L1, the rates of tumor growth inhibition (TGI) upon anti-PD1 therapy were 84.6% and 75.7%, respectively (Fig. 4A). Consistently, anti-PD1 treatments inhibited the growth of small-HIS-low tumors of LAP-pCDH and LAP-PD-L1 with TGIs of 49.5% and 57.4%, respectively (Fig. 4C). The TGI of large-HIS-low tumors of MCA38-PD-L1 was 71.9%, while the TGI of large-HIS-high tumors of MCA38-pCDH was 21.7% upon anti-PD1 therapy (Fig. 4B). Similarly, the TGIs of large-HIS-low tumors of LAP-PD-L1 and large-HIS-high tumors of LAP-pCDH were 44.0% and 4.4% upon anti-PD1 therapy, respectively (Fig. 4D). In large-HIS-low tumors of MCA38-PD-L1, when another immune checkpoint such as CTLA4 was targeted instead of the overexpressing one (PD-L1), the TGI was 20.6% (Supplementary Fig. S5), which was much lower than that of anti-PD1 therapy (Fig. 4B). These data collectively show that HIS-low tumors are sensitive to ICB therapy, while HIS-high tumors are resistant. Importantly, the HIS profiles can aid in selecting an appropriate immune checkpoint as the therapeutic target.

### Anti-PD1 therapy inhibits the growth of large tumor with HIS-low via CD8^+^ T cells

To explore the mechanism underlying the differential effects of anti-PD1 therapy on the growth of HIS-low and HIS-high tumors, we analyzed the compositions of intratumoral immune cells following treatments. In small-HIS-low tumors, anti-PD1 treatments increased the percentages of intratumoral CD4^+^ and CD8^+^ T cells while decreasing the proportions of immunosuppressive CD4^+^CD25^+^ T cells (Tregs) in both the MCA38-pCDH and MCA38-PD-L1 groups compared to their isotype-treated control groups (Supplementary Fig. S6). In large-HIS-low tumors of MCA38-PD-L1, anti-PD1 therapy significantly increased the percentages of intratumoral CD4^+^ and CD8^+^ T cells, along with their activated phenotypes CD4^+^CD44^+^CD69^+^ and CD8^+^CD44^+^CD69^+^, compared to the isotype control group. However, these alterations were not observed in the large-HIS-high tumors of MCA38-pCDH. Additionally, anti-PD1 treatments more profoundly decreased the proportions of Tregs in large-HIS-low tumors compared to large-HIS-high tumors. (Fig. 4E). To confirm whether T cells mediate the antitumor effects of anti-PD1 therapy in large-HIS-low tumors, we conducted T cell depletion experiments. In vivo depletion of CD4^+^ and CD8^+^ T cells simultaneously or CD8^+^ T cells alone did not influence tumor growth but completely reversed the tumor growth inhibition induced by anti-PD1 therapy (Fig. 4F). Collectively, these results suggest that CD8^+^ T cells mediate the antitumor effects of anti-PD1 therapy in large-HIS-low tumors.

### Large tumors with HIS-low contain fewer immunosuppressive cells

**Fig. 5:**
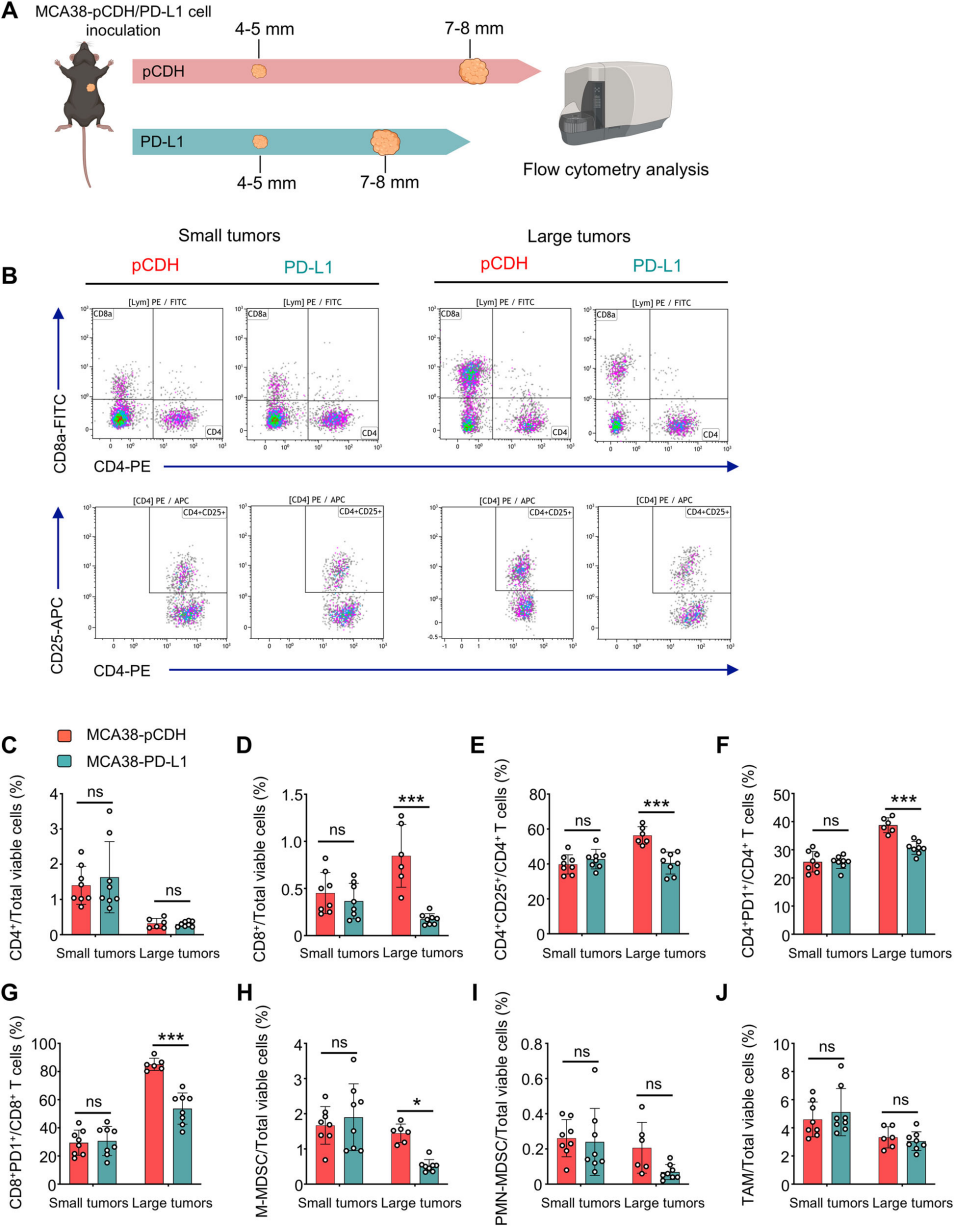
Large tumors with HIS-low harbored fewer immunosuppressive cells compared with large tumors with HIS-high. **A** MCA38-pCDH and MCA38-PD-L1 tumors were collected when they reached 4.0–5.0 mm and 7.0–8.0 mm in diameter, respectively. Intratumoral immune cells were analyzed by flow cytometry. **B** The representative flow cytometry plots of CD4^+^, CD8^+^ T cells, and CD4^+^CD25^+^ Tregs. The proportions of intratumoral CD4^+^ T cells (**C**), CD8^+^ T cells (**D**), Tregs (**E**), CD4^+^PD1^+^ T cells (**F**), CD8^+^PD1^+^ T cells (**G**), M-MDSCs (CD45^+^CD11b^+^Gr1^med^F4/80^med^, **H**), PMN-MDSCs (CD45^+^CD11b^+^Gr1^hi^F4/80^−^, **I**), and TAMs (CD45^+^CD11b^+^Gr1^−^F4/80^+^, **J**) were shown. Data were from one experiment representative of two independent experiments with similar results. Each dot represents one individual tumor sample. Data are shown as the mean ± SEM and were analyzed by two-way ANOVA.

Given the significantly better response of large-HIS-low tumors to anti-PD1 therapy, we inferred that the composition of immune cells differs between large-HIS-low and -high tumors. To test this, we analyzed the intratumoral immune cell compositions in large-HIS-low and -high tumors **(**Fig. 5A**)**. The proportions of CD8^+^ T cells, Tregs, and monocytic myeloid-derived suppressor cells (M-MDSCs), along with the fractions of CD4^+^PD1^+^ and CD8^+^PD1^+^ T cells were all reduced in the large-HIS-low tumors compared to those of large-HIS-high tumors. However, in the context of small-HIS-low tumors for both MCA38-pCDH and MCA38-PD-L1, the percentages of these immune cell populations were comparable (Fig. 5). Taken together, the data suggest that large-HIS-low tumors harbor fewer immunosuppressive cell components, resulting in a more immuno-supportive tumor microenvironment that may facilitate anti-PD1 therapy.

### PD-L1 more reliably predicts anti-PD1 response in patients with baseline large tumors

The above preclinical data show that baseline small tumors are consistently characterized as HIS-low regardless of PD-L1 expression levels. Conversely, in baseline large tumors, high levels of PD-L1 expression result in HIS-low and sensitivity to anti-PD1 therapy. This led us to investigate whether the predictive performance of PD-L1 TPS is influenced by baseline tumor sizes in clinical settings.

In a randomized, double-blind, phase III study of nonsquamous NSCLC evaluating the efficacy of the combination of sintilimab (an anti-PD1 antibody from Innovent Biologics) and chemotherapy (pemetrexed and platinum) (ClinicalTrials.gov: NCT03607539) [29], we screened the CECT images of 397 patients and obtained 129 patients receiving anti-PD1 combination therapy whose CECT images were eligible for tumor volume analysis. The cutoff value of BTV with the highest Youden index was 10575.21 determined by the ROC curves. Patients in the BTV-small or -high subgroups had comparable baseline clinical characteristics, such as age, Eastern Cooperative Oncology Group (ECOG) scores, smoking history, stages, brain/liver metastasis, and the levels of PD-L1 expression (Table 1).

**Table 1:** The clinical characteristics of the NSCLC cohort.

Patient characteristics	Small tumors (*n* = 37)	Large tumors (*n* = 92)	Total cohort (*n* = 129)
Age, median (range)	60.4 (37, 74)	59.6 (39, 72)	59.8 (37, 74)
≥60 years, *n* (%)	21 (56.8)	50 (54.3)	71 (55.0)
Sex, *n* (%)			
Male (%)	23 (62.2)	75 (81.5)	98 (76.0)
Female (%)	14 (37.8)	17 (18.5)	31 (24.0)
ECOG score: *n* (%)			
0	8 (21.6)	28 (30.4)	36 (28.0)
1	29 (78.4)	64 (69.6)	93 (72.0)
Smoking history			
Smoker	21(56.8)	65(70.7)	86(66.7)
Never smoker	16(43.2)	27(29.3)	43(33.3)
Pathology			
Adenocarcinoma	36(97.3)	89(96.7)	125(96.9)
Other	1(2.7)	3(3.3)	4(3.1)
Clinical stage			
IIIb/IIIc	2(5.4)	7(7.6)	9(7.0)
IV	35(94.6)	85(92.4)	120(93.0)
Brain metastasis			
Yes	6(16.2)	15(16.3)	21(16.3)
No	31(83.8)	77(83.7)	108(83.7)
Liver metastasis			
Yes	3(8.1)	16(17.4)	19(14.7)
No	34(91.9)	76(82.6)	110(85.3)
Response: *n* (%)			
Responders	26 (70.3)	51 (55.4)	77 (59.7)
Non-responders	11 (29.7)	41 (44.6)	52 (40.3)
PD-L1 TPS (%): *n* (%)			
<1	13 (35.1)	30 (32.6)	43 (33.3)
≥1	24 (64.9)	62 (67.4)	86 (66.7)
≥25	15 (40.5)	48 (52.2)	63 (48.8)
<50	28 (75.7)	51 (55.4)	79 (61.2)
≥50	9 (24.3)	41 (44.6)	50 (38.8)
≥75	4 (10.8)	30 (32.6)	34 (26.4)

The number of patients with the given characteristic is shown. The numbers in parentheses represent the percentages of indicated patients.

*NSCLC* non-small cell lung cancer, *ECOG* Eastern Cooperative Oncology Group, *PD-L1* programmed death ligand 1, *TPS* tumor proportion score.

**Fig. 6:**
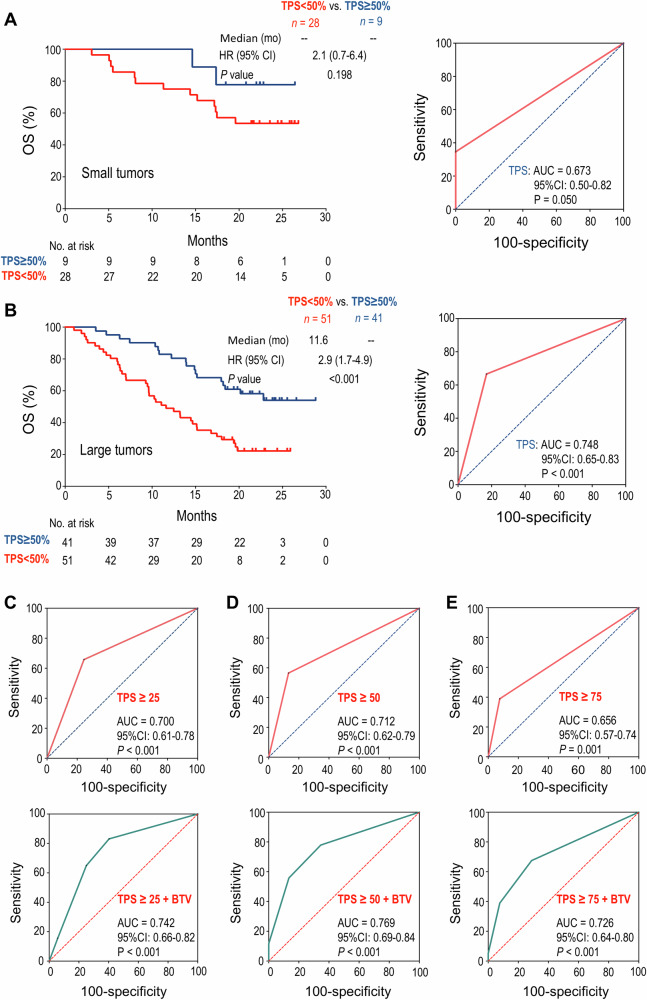
A bivariate model of PD-L1 TPS and BTV robustly predicted response to anti-PD1 combination therapy in NSCLC patients. **A** In the patient subgroup with small BTV, Kaplan–Meier analysis of OS stratified by baseline PD-L1 TPS ≥ 50% or < 50% (left), and ROC curve of PD-L1 TPS to predict response to anti-PD1 combination therapy (right). **B** In the patient subgroup with large BTV, Kaplan–Meier analysis of OS stratified by baseline PD-L1 TPS ≥ 50% or <50% (left), and ROC curve of PD-L1 TPS to predict response to anti-PD1 combination therapy (right). **C**–**E** The ROC curves for PD-L1 TPS alone (up) or in combination with BTV (down) for predicting response to anti-PD1 combination therapy. **C** PD-L1 TPS ≥ 25%, **D** PD-L1 TPS ≥ 50%, **E** PD-L1 TPS ≥ 75%. The difference between survival curves was determined by log-rank tests, and the AUCs were analyzed by DeLong’s tests. BTV baseline tumor volume, OS overall survival, TPS tumor proportion score, ROC receiver operating characteristic, AUC area under the curve, HR hazard ratio, CI confidence interval.

In the BTV-small subgroup (BTV ≤ 10575.21), PD-L1 TPS ≥ 50% was not significantly associated with longer OS (HR: 2.1, 95% CI: 0.7–6.4, *P* = 0.198) (Fig. 6A). Correspondingly, the AUC for PD-L1 TPS in predicting tumor response to anti-PD1 combination therapy was 0.67 (*P* = 0.05, Fig. 6A). In contrast, in the BTV-large subgroup, PD-L1 TPS ≥ 50% exhibited a significant association with longer OS (HR: 2.9, 95% CI: 1.7–4.9, *P* < 0.001) compared to PD-L1 TPS < 50%, and the AUC for PD-L1 TPS in predicting therapeutic response was 0.75 (*P* < 0.001, Fig. 6B). These results show that PD-L1 TPS more reliably predicts therapeutic response in NSCLC patients with baseline large tumors.

### A bivariate model of PD-L1 TPS and BTV robustly improves predictive performance

Next, we conducted further analysis to establish a bivariate prediction model of PD-L1 TPS and BTV. Consistent with the clinical data showing that different levels of PD-L1 TPS were associated with differential therapeutic efficacy [29], the AUCs for predicting patient response to anti-PD1 combination therapy were 0.70, 0.71, or 0.66 when PD-L1 TPS was ≥25%, 50% or 75%, respectively (all with *P* ≤ 0.001). The predictive sensitivity was 64.9%, 55.8%, 39.0% and the specificity was 75.0%, 86.5%, and 92.3%, corresponding to the cut-off values of PD-L1 TPS ≥ 25%, 50%, 75%, respectively (Fig. 6C–E). Remarkably, in a bivariate model combining BTV and PD-L1 TPS ≥ 25%, 50%, or 75%, the AUCs for predicting the therapeutic responses were 0.74, 0.77, or 0.73, respectively (all with *P* < 0.001), accompanied by the predictive sensitivity of 83.1%, 77.9%, 67.5%, and the specificity of 59.6%, 65.4%, 71.2%, respectively (Fig. 6C–E). Taken together, these results suggest that the integration of PD-L1 TPS ≥ 50% with baseline tumor volume substantially improves the predictive accuracy in NSCLC patients.

## Discussion

ICB therapy has revolutionized advanced cancer treatments over the last decade, yet only a minority of patients obtained long-term survival benefits [20, 21, 35]. Despite exploration of various combination strategies and biomarkers to fortify ICB therapy, the improvements have been limited. Our study revealed that the heterogeneity of immune checkpoint profiles within the TME was closely linked to the selective efficacy of ICB therapy. Tumor progression increased the HIS, which was associated with resistance to anti-PD1 therapy. Interestingly, PD-L1 high-expression hindered the upregulation of a majority of other immune checkpoint genes during tumor progression, leading to decreased HIS levels and increased sensitivity to ICB therapy. These preclinical findings suggest that the resistance of large tumors to ICB therapy is mediated by increased HIS. Consistently, a retrospective analysis of a phase III study in NSCLC patients showed that PD-L1 TPS ≥ 50% more reliably predicted anti-PD1 responses in patients with baseline large tumors. Remarkably, a bivariate model of PD-L1 TPS and BTV significantly improved predictive reliability. These findings highlight the importance of the heterogeneity of immune checkpoint signature in tumor immune evasion and suggest potential new strategies to optimize patient stratification and ICB therapy according to the profiles of HIS.

**Fig. 7:**
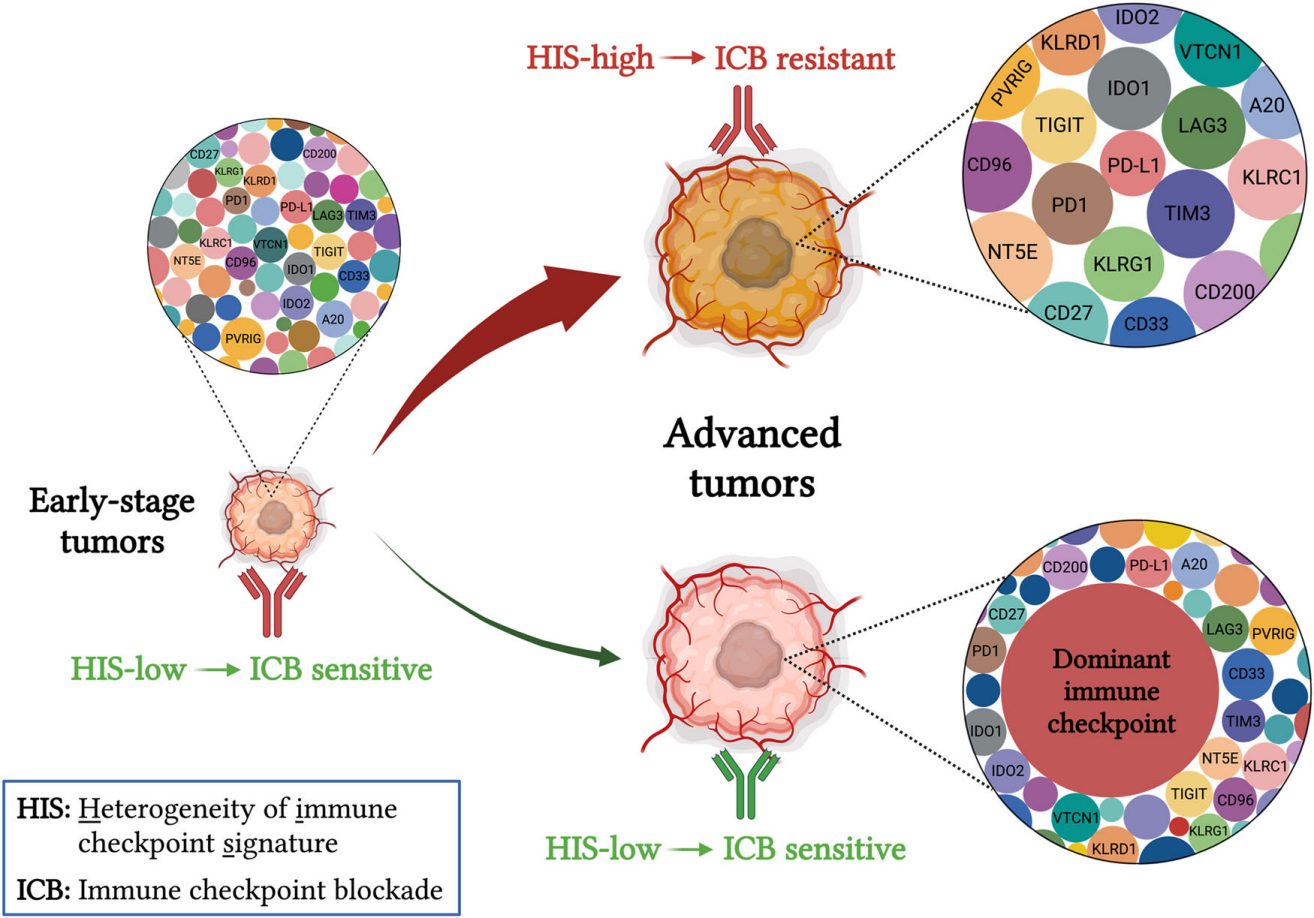
A proposed model shows that HIS reflects the dynamic features of tumor immune evasion and dictates the selective efficacy of ICB in a tumor size-dependent manner. During early tumorigenesis, immune checkpoints are usually expressed at relatively low levels (HIS-low), thus sensitive to ICB therapy. Following tumor progression, the TME becomes increasingly immune suppressive to evade host immunosurveillance. This process involves two potential mechanisms: a stochastic upregulation of multiple immune checkpoints, leading to HIS-high and resistance to ICB therapy (Majority), or overexpression of a few pivotal immune checkpoints while maintaining others at lower levels (Minority). Therefore, the elevated expression of key immune checkpoints reduces the HIS in large tumors compared to small tumors, rendering those large tumors sensitive to blocking the predominant immunosuppressive signal in the TME. ICB immune checkpoint blockade, HIS heterogeneity of immune checkpoint signature.

The initiation and progression of tumors rely on their capacity to evade host immunosurveillance [36–38]. Larger tumors usually harbor a more pronounced immunosuppressive milieu compared to smaller ones [39–41]. Indeed, smaller tumors often exhibit greater sensitivity to immunotherapy in both preclinical and clinical settings. In larger tumors, the extent of immunosuppression is contingent upon the quantity and intensity of immunosuppressive factors. Tumor progression generally leads to random upregulation of multiple immune checkpoint molecules (HIS high), a phenomenon often linked to resistance to PD1/PD-L1 blockade therapy (Fig. 7). In an alternate scenario, when a tumor possesses a predominant immunosuppressive factor that is adequate for enabling the tumor to evade host immunosurveillance (HIS low), the tumor does not necessitate the induction of additional immunosuppressive factors for its continued progression. Such tumors would likely be sensitive to the blockade of the predominant immunosuppressive factor (Fig. 7). In the natural process of tumor evolution, the majority of tumors enhance immune evasion capability through increasing HIS, while only a minority evolve into a HIS-low mechanism (Fig. 7). This may explain why the long-term efficacy of ICB therapy is limited to a small subset of patients (<30%) in clinical practice [20, 21, 35]. Moreover, the findings of this study suggest that the application of ICB therapy to earlier stages of cancer patients may achieve greater benefits because they are often HIS-low. It is interesting that recent clinical data suggest that CRC patients with either deficient mismatch repair (dMMR)/microsatellite instability-high (MSI-H) or proficient mismatch repair (pMMR)/microsatellite stability (MSS) could obtain more benefits from ICB therapy if they receive ICB treatments at earlier stages [42].

The low response rate of ICB therapy has spurred efforts to explore more reliable strategies for stratifying potentially sensitive patients [43]. The concept of HIS could enhance patient stratification. Although PD-L1 TPS has been approved as a companion predictive biomarker for immunotherapy, its predictive performance remains suboptimal [44, 45]. Firstly, diverse cut-off thresholds of PD-L1 TPS have been used to predict patient response to PD-L1/PD1 blockade therapy, making the determination of the optimal TPS threshold elusive. According to HIS, higher TPS would correspond to lower HIS and increased specificity. In the phase III (NCT03607539) study, the predictive specificity reached nearly 100% when TPS ≥ 75%, but the corresponding sensitivity was only 39.0%. Hence, to achieve a balance between the predictive specificity and sensitivity, the median values of TPS, such as 30–50%, would be optimal. Secondly, TPS and the combined positive score (CPS) [46], which one could be better? TPS and CPS reflect the relative expression levels of PD-L1 in tumor cells alone or collectively in tumor cells, lymphocytes and macrophages, respectively [47]. Both TPS and CPS have been used to predict anti-PD1 responses. The disparate viewpoints exist regarding which part of PD-L1 corresponds to PD-L1/PD1 blockade therapy. According to the concept of HIS, the pivotal concern appears to be the magnitude of PD-L1 expression rather than the specific location. Lastly, an interesting finding of HIS is that overexpressing one immune checkpoint, such as PD-L1, selectively reduced HIS in large, but not small, tumor. Thus, a cost-effective strategy to improve PD-L1 prediction is to integrate it with baseline tumor volume. Indeed, our retrospective analysis showed that a bivariate model of TPS and BTV robustly predicted patient response to anti-PD1 combination therapy in NSCLC.

The concept of HIS may guide to the development of more effective ICB combination therapies to improve response rates. Currently, commonly used combination immunotherapies include PD1/PD-L1 blockade combined with another ICB, such as anti-CTLA4, or with another cancer treatments, such as chemotherapy and antiangiogenic therapy [20, 48]. However, since large tumors usually exhibit increased expression of multiple immune checkpoints, blocking one or two immune checkpoints may not be sufficient to induce potent antitumor immunity. The combination of ICB with another therapeutic strategy to reduce HIS may help to overcome drug resistance. For tumors with overexpression of pivotal immune checkpoints, the profile of HIS may help to choose immune checkpoint targets. For instance, in the context of MCA38-PD-L1 large tumors, the levels of PD-L1, Nt5e, Tdo2, and CD33 were increased compared with their small tumors. Therefore, combination therapy targeting PD1 along with the upregulated immune checkpoints may optimize treatment efficacy. Furthermore, given the heterogeneity of tumors, the profiles of HIS vary among patients, underscoring the importance of individualized treatment strategies based on the distinct HIS characteristics.

Our current study also has limitations. First, the subcutaneous preclinical tumor models may not fully recapitulate the complexity of the human tumor microenvironment. Second, this study used only two preclinical tumor models with PD-L1/PD1 blockade therapy. Therefore, further validation of our conclusions in additional tumor types and with other immune checkpoint inhibitors in both preclinical and clinical settings is essential to establish the generalizability of our findings. Third, the gene set used for HIS analysis is based on currently known immune checkpoint molecules, which likely only represent a fraction of those present in the TME. When new immune checkpoints are identified, our findings will need to be re-evaluated. Fourth, our initial understanding of HIS was derived from bulk tumor tissue analyses. Since immune checkpoints can be expressed differentially on immune cells, other stromal cells, and tumor cells, our future work will analyze and compare the impacts of HIS derived from bulk tumor tissues and specific cell populations on ICB efficacy. Gaining a deeper understanding of the specific impacts of HIS across different cell populations might pave the way for developing personalized therapeutic approaches tailored to specific tumor types. Our preliminary single-cell RNA sequencing analysis revealed that immune checkpoint molecules are predominantly expressed on T cells, myeloid cells, and fibroblasts (Supplementary Fig. S7), suggesting that these cell populations may serve as key determinants of HIS characteristics. Finally, the clinical translation of the combination of immune checkpoint biomarkers with BTV has its limitations. For PD-L1 to serve as a reliable biomarker, its baseline levels should be sufficiently high. In tumors with inherently low PD-L1 expression, the PD1/PD-L1 pathway likely plays a subordinate role in immune evasion. Moreover, in the early-stage cancers undergoing neoadjuvant immunotherapy, the combination of PD-L1 with BTV may not improve prediction because tumor sizes are relatively small. Additionally, the bivariate model may not improve prediction in cancers with low mutation burden, such as brain cancer and pancreatic cancer.

In conclusion, our study suggests that the HIS profile could determine the outcomes of ICB therapy. The concept of HIS not only suggests the application of ICB to earlier stages of cancer patients but also offers a novel approach to tailor personalized ICB therapy. In addition, we found that high levels of PD-L1 expression resulted in reduced HIS in large tumors, sensitizing them to anti-PD1 therapy. This finding offers a promising and readily applicable avenue for enhancing the predictive potency of PD-L1 in clinical settings through integrating baseline tumor volume into the analysis. In the future, the concept of HIS could be applied to other immunosuppressive factors beyond immune checkpoints.

## Supplementary information

Supplementary figures and table

Patient information

Daw data of RNA-seq and qPCR

## Data Availability

All data supporting the findings of this study are available within the Article and its Supplementary Information. Bulk RNA-seq data have been deposited in the NCBI BioProject database under accession number PRJNA1198210. The single-cell RNA sequencing data for CRC and NSCLC are available from the Tumor Immunotherapy Gene Expression Resource (TIGER) database at http://tiger.canceromics.org.
