# Supplementary material for: High baseline levels of PD-L1 reduce the heterogeneity of immune checkpoint signature and sensitize anti-PD1 therapy in lung and colorectal cancers
Source: Cell Death Dis. 2025 Mar 4;16(1):152. doi: 10.1038/s41419-025-07471-w (PMC11880386; doi:10.1038/s41419-025-07471-w)
Supplement: Supplementary file 1 — Supplementary figures and table [file 41419_2025_7471_MOESM1_ESM.pdf]

Supplemental material

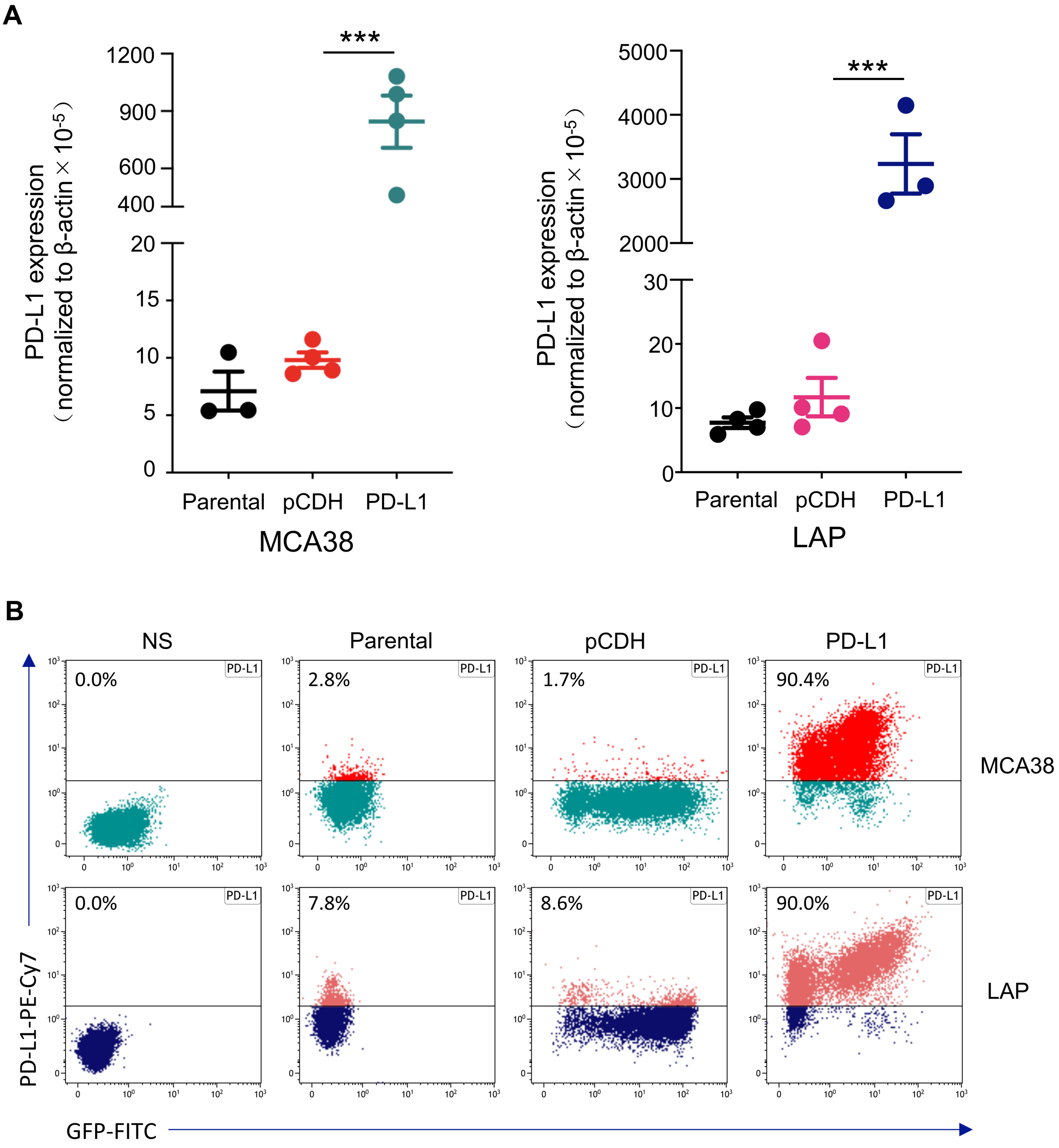

Fig. S1 MCA38 and LAP cell lines with PD-L1 overexpression.

(**A**) The transcription of *Pd-l1* in MCA38 and LAP tumor cells was determined by qPCR. The experiments were repeated three times independently (n = 3-4). Data are showed as the mean ± s.e.m and were analyzed by one-way ANOVA. (**B**) The representative flow cytometry plots showed PD-L1 expression on MCA38 and LAP tumor cells.

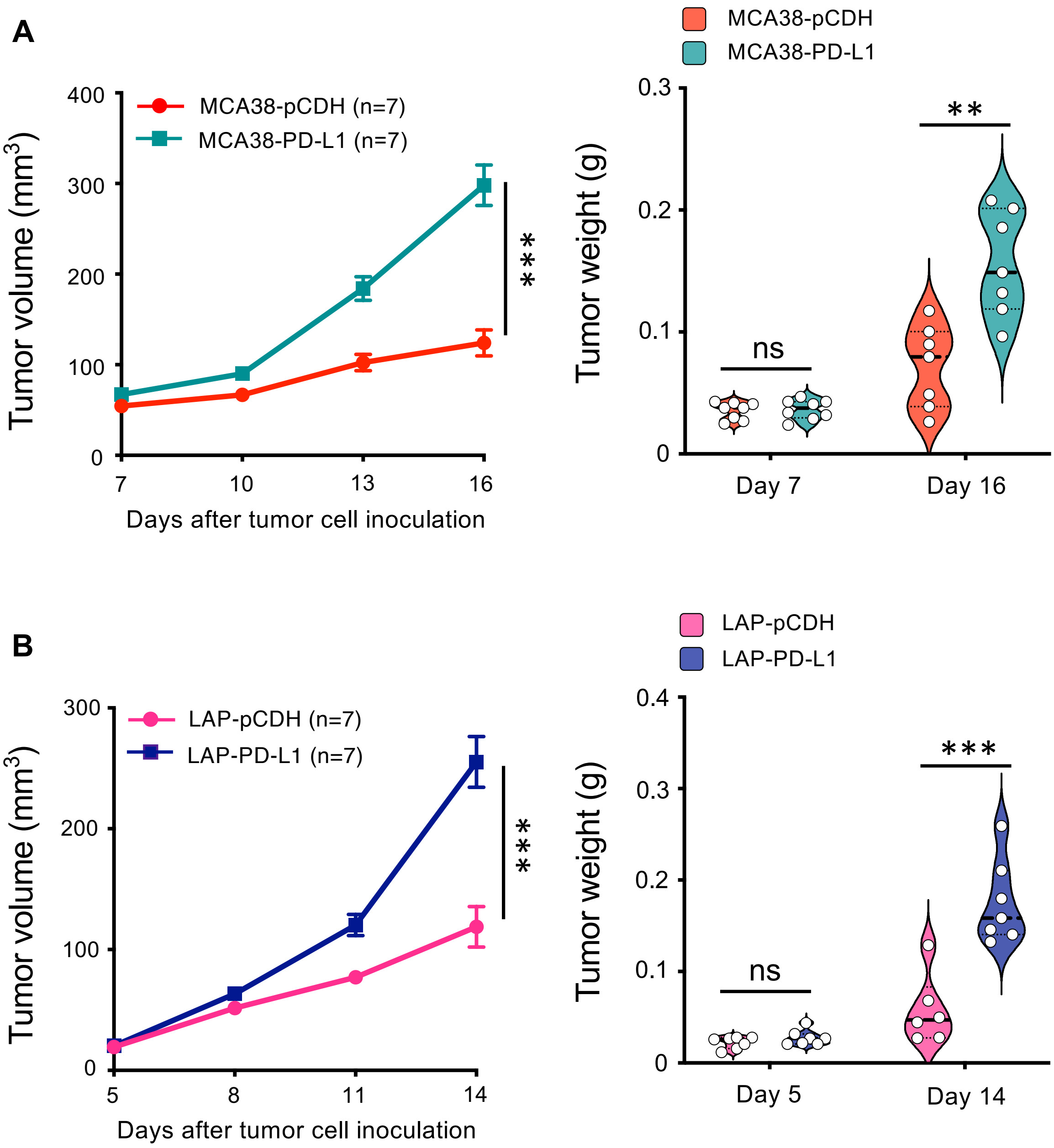

Fig. S2 Overexpressing PD-L1 in MCA38 and LAP tumor cells promoted late-stage tumor growth.

(**A**) The tumor growth curves of MCA38-pCDH and MCA38-PD-L1 and the tumor weights harvested on days 7 and 16 post-inoculation, respectively. (**B**) The tumor growth curves of LAP-pCDH and LAP-PD-L1 and the tumor weights harvested on days 5 and 14 post-inoculation, respectively. Data were from one experiment representative of three independent experiments with similar results. Each dot represents one individual tumor. Data are shown as the mean ± s.e.m and were analyzed by two-tailed student's *t*-tests.

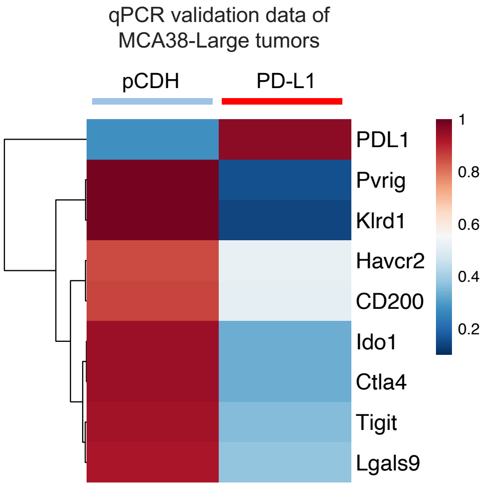

Fig. S3 High PD-L1 expression corresponded to reduced HIS in large MCA38 tumors.

qPCR analysis to validate the results of RNA-seq in large MCA38-pCDH and MCA38-PD-L1 tumors. The corresponding heatmap showed group-level representations, with three biological replicates (n = 3).

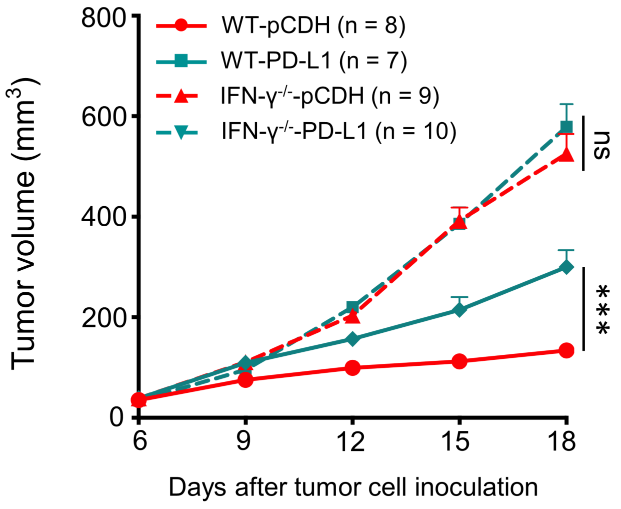

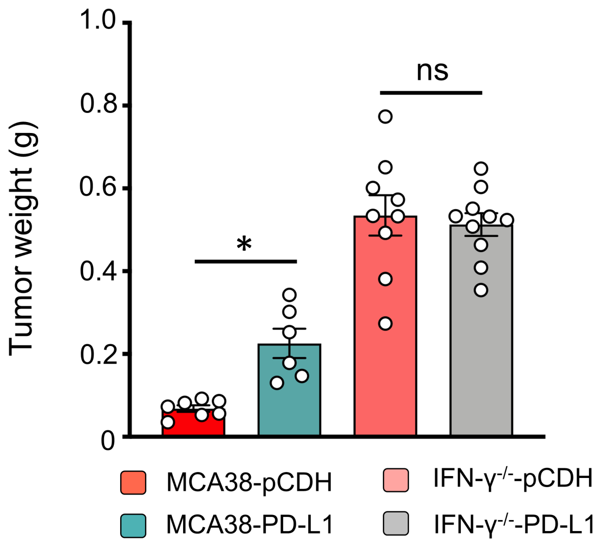

Fig. S4 *Ifng* Deficiency abrogated the pro-tumoral effects of PD-L1 overexpressing in MCA38 tumors.

WT and *Ifng* knockout mice were subcutaneously injected with 2×10^5 MCA38-pCDH or MCA38-PD-L1 tumor cells. Tumor volumes were monitored every three days, and tumor weights were recorded at the time of tumor collection. The significance was determined by two-way ANOVA. Each dot represents one individual tumor. The data are presented as the mean ± s.e.m.

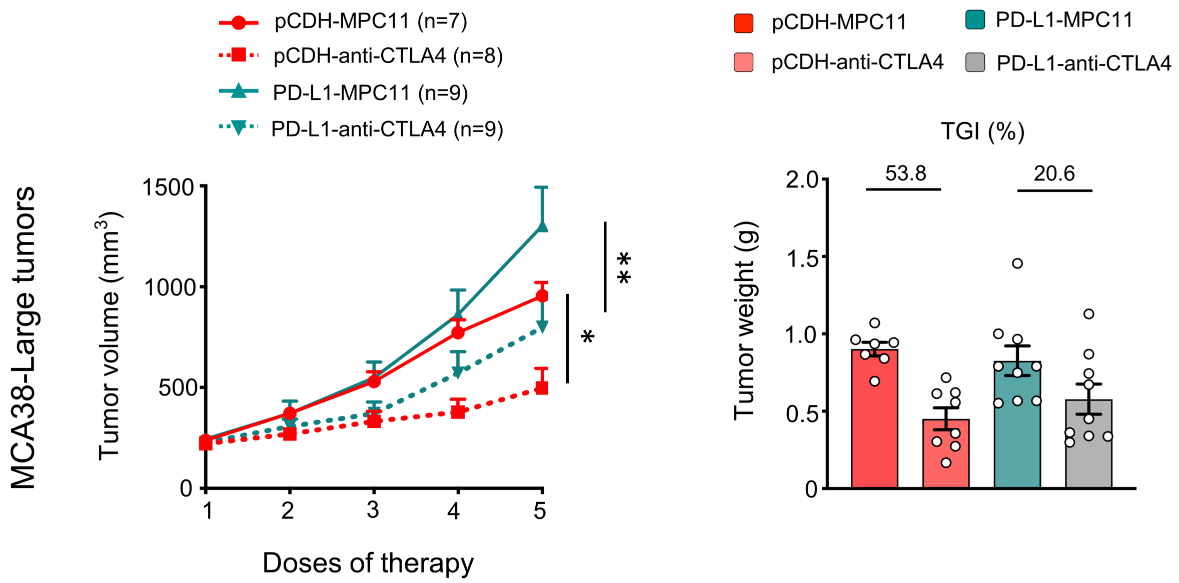

Fig. S5 Anti-CTLA-4 treatment was less effective in large MCA38-PD-L1 tumors compared to MCA38-pCDH.

The growth curves and tumor growth inhibition (TGI) of MCA38-pCDH and MCA38-PD-L1 tumors in female C57BL/6 mice treated with either IgG_{2b} or an anti-CTLA4 antibody. Treatments were initiated when tumor size reached 7.0-8.0 mm in diameter. The significance was determined by two-way ANOVA. Each dot represents one individual tumor. The data are presented as the mean ± s.e.m.

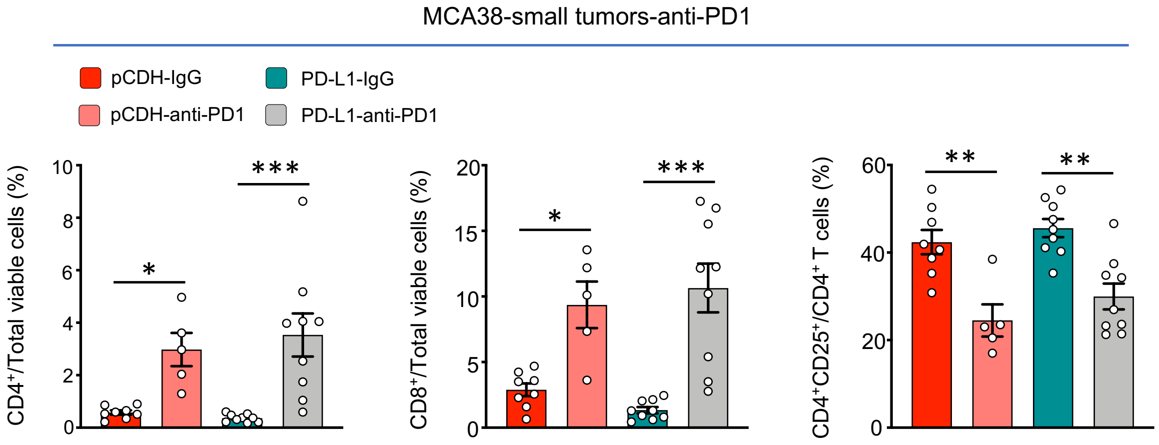

Fig. S6 Anti-PD1 therapy increased the proportions of intratumoral T cells while reducing Tregs in HIS-Low tumors.

Control MCA38-pCDH or MCA38-PD-L1 tumors were treated with anti-PD1 or isotype IgG when tumors reached 4.0-4.5 mm and continued every 3 days. The percentages of intratumoral $CD4^+$ T cells, $CD8^+$ T cells, and $CD4^+CD25^+$ Tregs were analyzed by flow cytometry. Data were from one experiment representative of two independent experiments with similar results. Each dot represents one individual tumor. Data are shown as the mean ± s.e.m and were analyzed by two-way ANOVA.

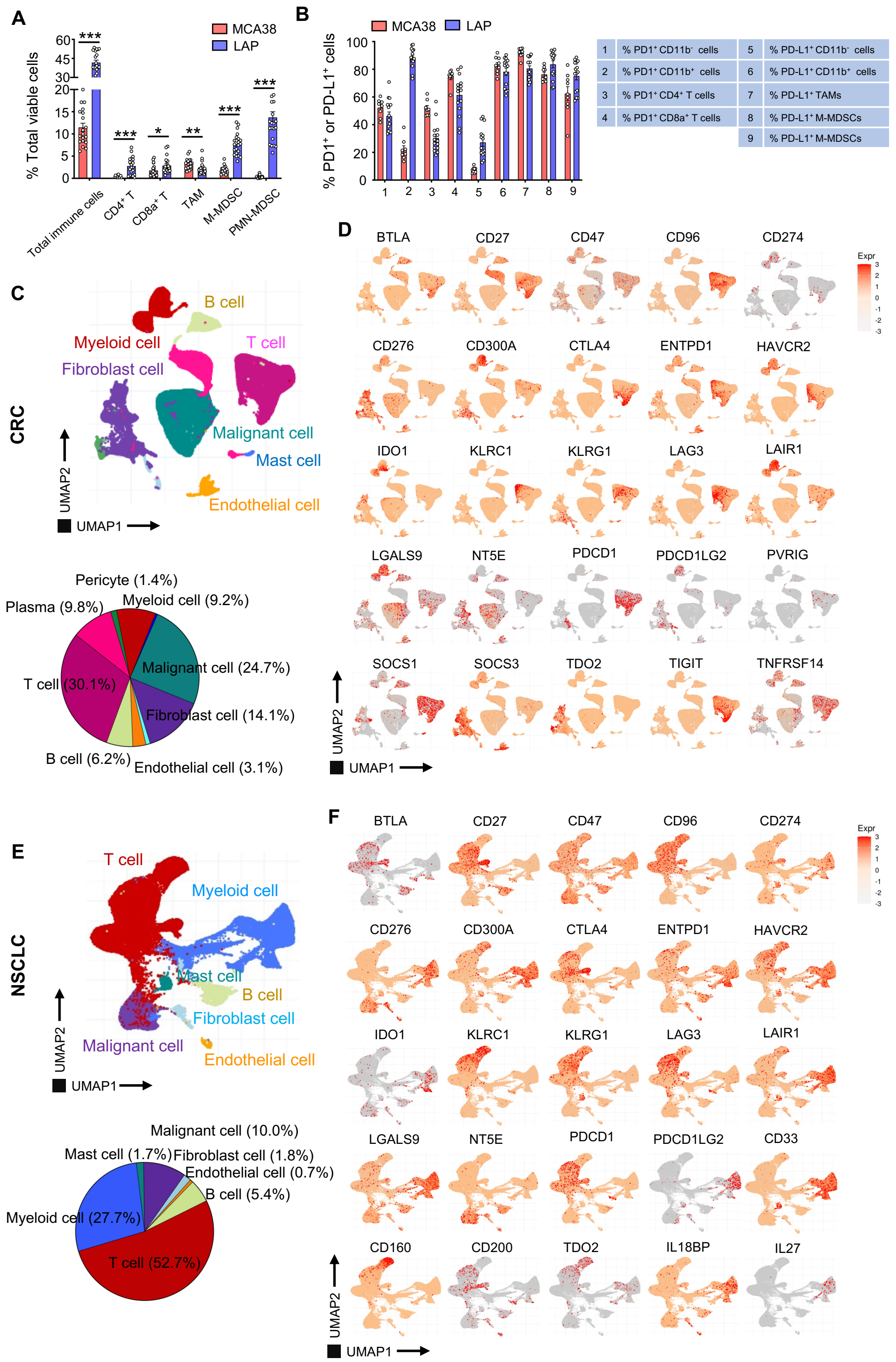

Fig. S7 Comparative analysis of immune cell composition and immune checkpoint expression in the tumor immune microenvironment of colorectal and lung cancers.

(**A**) The proportions of immune cell populations in murine MCA38 colorectal and LAP0297 lung tumor models. Each dot represents one individual mouse. Data are shown as the mean ± s.e.m and

were analyzed by two-tailed Student's *t*-tests. (**B**) Flow cytometry analysis of PD1 expression on $CD45^{+}CD11b^{-}$ cells, $CD45^{+}CD11b^{+}$ cells, $CD4^{+}$, and $CD8^{+}$ T cells, and PD-L1 expression on $CD45^{+}CD11b^{-}$ cells, $CD45^{+}CD11b^{+}$ cells, tumor-associated macrophages (TAMs), monocytic myeloid-derived suppressor cells (M-MDSCs), and polymorphonuclear myeloid-derived suppressor cells (PMN-MDSCs) in murine MCA38 colorectal and LAP0297 lung tumor tissues. (**C-F**) The analysis of single-cell RNA sequencing data from colon cancer (CRC) and non-small cell lung cancer (NSCLC) patient samples using the tumor immunotherapy gene expression resource (TIGER) database[1, 2]. (**C**) Uniform Manifold Approximation and Projection (UMAP) plots showing the cellular clusters in pre-treatment CRC tumor tissue（up）and the proportions of each cell population (down). (**D**) The expression of immune checkpoints in CRC tissues. (**E**) UMAP plots showing the cellular clusters in pre-treatment NSCLC (up) and the proportions of each cell population (down). (**F**) Immune checkpoint expression in NSCLC tissues.

References

1. Lee HO, Hong Y, Etlioglu HE, Cho YB, Pomella V, Van den Bosch B*, et al.* Lineage-dependent gene expression programs influence the immune landscape of colorectal cancer. *Nat Genet* 2020, **52**(6)**:** 594-603.

2. Laughney AM, Hu J, Campbell NR, Bakhoum SF, Setty M, Lavallee VP*, et al.* Regenerative lineages and immune-mediated pruning in lung cancer metastasis. *Nat Med* 2020, **26**(2)**:** 259-269.

Table S1
List of the sequences of qPCR primers

Gene	Forward primer (5'-3')	Reverse primer (5'-3')
B-actin	ATGGAAAATTATACTCAGTGGCTGC	ATTTAGTACCTGGGTCTCAACAT
Pd-l1	TCACGGCTCCAAAGGACTTG	TGATTCGCTTGTAGTCCGCA
Havcr2	ACTGGTGACCCTCCATAATAACA	GCAGTTCTGATCGTTTCTCCA
Nt5e	CGCTCAGAAAGTTCGAGGTGTG	CGCAGGCACTTCTTTGGAAGGT
Lag3	CTCCATCACGTACAACCTCAAGG	GGAGTCCACTTGGCAATGAGCA
Tigit	TCCAAGAAAGCTCAGTGGCTCAG	GAGAGACTCCTCAGGTTCCATTC
Pdcd1lg2	CTGGGACTACAAGTACCTGACG	CTCTAGCCTGGCAGGTAAGCTG
Pvrig	ACCCTCTGTGTCTCTGAAGCAAG	AGTTTGGTCCTTCCAGCGT
Vtcn1	GTTACATCCGCTCCTCAAAAGGC	GCAGCGTAAACTCTCTGAACTGG
Ido1	GCAGACTGTGTCCTGGCAAACT	AGAGACGAGGAAGAAGCCCTTG
Tnfaip3	AGCAAGTGCAGGAAAGCTGGCT	GCTTTCGCAGAGGCAGTAACAG
Cd33	GCTCCTGATTCTTGGGCTCT	ATCGCTTCCTGCCTCTTGATAG
Lair1	AGACCCATCCACGTCCCATA	ACTGGGGATGTAAAGGCAGC
Lgals9	CTGGAATCCCTCCTGTGGTGTA	CCTCGTAGCATCTGGCAAGACA
Ctla4	GTACCTCTGCAAGGTGGAACTC	CCAAAGGAGGAAGTCAGAATCCG
Cd300a	CTGGAGGATGCAGACACCTACA	CTCTAGTGTTGGTCCTGGAGATG
Cd155	GAGGCAGTAGAAGCACCAATGC	GGTGACCATTGGCAGAGATGCA
Tdo2	CATGCTCAAGGTGATAGCTCGG	GGAAGCCTGATGCTGGAGACAG
Cd276	AGCATCCAGGACTTTGACAGCG	CGTGATGGTCACCATGTTCCCT
Cd160	GCCACTTTCTCTCCGTTCTAGTC	AGGAAGCCTGAACTGAGAGTGC
Cd200	GAAGGTCTCAGGAACAGCTTGC	GCAGTCGCAGAGCAAGTGATGT
Cd200r	TGTGAGACAGTAACACCTGAAGG	TGCCATTGCCTCACAGACTGCA
Ido2	GACAGTCTTGGTGGAGAAGGCA	ATCCTGGATGGAGAGTCTCAGC
Vista	AAACCACCACCCAGAACAACGG	ACTGTCCTGCTCATTAGACGCC
Il18bp	TCTCCAGCAGTCCCAACTAAGC	AGGCAGTACAGGACAAGGTCAG
Cd112	GCCATACTGACCTGTGATGTACG	TCCACAGAGTGGACAAGCAGCT
Klrc1	CTCATGGACTGGAATCCTTCGG	GACCAGAAGCTGACATCATGGC
Klrb1b	ACCGTTAACACATTTCCAGAATGC	AGTTTCAGAGCCACCCGATG
Entpd1	CTGGACAAGAGGAAGGTGCCTA	GACTGTCTGAGATGAGGCTTAGC
Socs1	AGTCGCCAACGGAACTGCTTCT	GTAGTGCTCCAGCAGCTCGAAA
Cd96	CATGACAGCTTGGTGTATGGCTC	CAGTGGGTAGATGTTTCGTTGGG
Btla	GCTTGGGACTCCTCGGTTAT	GCACTGGACACTCTTCATCATT
Pdcd1	CGGTTTCAAGGCATGGTCATTGG	TCAGAGTGTCGTCCTTGCTTCC
